# DNA analysis of the Russian populations of Aberdeen Angus, Hereford and Belgian Blue cattle

**DOI:** 10.5194/aab-63-409-2020

**Published:** 2020-11-16

**Authors:** Elena N. Konovalova, Olga S. Romanenkova, Valeria V. Volkova, Olga V. Kostyunina

**Affiliations:** Laboratory of the selection molecular basis, L.K. Ernst Federal science Center for Animal Husbandry, Dubrovitsy, Podolsk district, Moscow region, 142132, Russia

## Abstract

The use of specialized meat breeds in cattle breeding programs is considered very promising for improving herds' productivity. However, in animal genotype, along with genes that positively affect the productivity signs, there are genes whose mutations, known as genetic defects, negatively affect the health of animals. The aim of the study was the screening of the Russian populations of Aberdeen Angus, Hereford and Belgian Blue cattle on gene mutations associated with the genetic defects of arthrogryposis multiplex (AM), osteopetrosis (OS), developmental duplication (DD), double muscling (M1), hypotrichosis (HY) and maple syrup urine disease (MSUD) as well as the *F94L* polymorphism of myostatin gene (*MSTN*) linked with the gene responsible for less fat content in the carcass by means of DNA analysis. In the article, test systems based on the polymerase chain reaction method are presented. The analysis of the Aberdeen Angus (n=4480) population has revealed 0.19 ± 0.09 % animal M1 carriers, 0.53 ± 0.03 % OS carriers, 1.92 ± 0.09 % AM carriers and 9.00 ± 0.20 % DD carriers. The genotyping of Hereford cattle of Russian populations (n=525) has not revealed any individual carriers of MSUD or HY genetic defects. All of the Belgian Blue population (n=92) animals were heterozygous M1 carriers. The study of the *F94L* *MSTN* polymorphism has demonstrated extremely high frequencies of the desirable A allele (0.93 and 0.90) in two Aberdeen Angus populations with an average mean of 0.63 ± 0.08, which was 32 % higher compared to the Belgian Blue population. The results suggest the high genetic potential of the Aberdeen Angus and Belgian Blue cattle, but the existence in the genotypes of the mutant alleles associated with hereditary diseases indicates the risk of uncontrolled use of these breeds.

## 1 Introduction

The use of breeding material of specialized meat cattle breeds is very promising for improving beef cattle breeding profitability (Dusaeva and Kuvanov, 2013). Excellent productive features such as high meat yield and good organoleptic traits due to increased tenderness and marbling were registered among the animals of the Aberdeen Angus, Hereford and Belgian Blue breeds (Ragimov et al., 2019). Also, the animals of these breeds have good acclimatization abilities and high genetic potential (Ragimov et al., 2019; Urioste et al., 2007). However, besides the advantages, Aberdeen Angus, Hereford and Belgian Blue cattle have a serious drawback, i.e., the appearance of some genetic defects (Gholap et al., 2014).

According to the American Angus Association, today in the Aberdeen Angus cattle breed there is an appearance of a number genetic anomalies, among them arthrogryposis multiplex (AM), osteopetrosis (OS), developmental duplication (DD) and double muscling (M1). The clinical features of these anomalies were described in previous studies (Gholap et al., 2014; Windsor et al., 2011). The focus of our interest are the mutations caused. It should also be noted that AM and OS are the lethal genetic defects leading to calf deaths, and DD and M1 are non-lethal but undesirable conditions.

The reason for AM is the large deletion (23 347 bp) encompassing three genes: the completely ISG15 ubiquitin-like modifier (*ISG15*) gene, the 5${}^{\prime }$ regulatory region of the hairy and enhancer split 4 (*HES4*) and two first exons of the agrin gene (*AGRN*) (Windsor et al., 2011).

The reason for OS is the deletion of 2784 bp encompassing completely exon 2 and half of exon 3 of solute carrier family 4 (anion exchanger) member 2 gene (*SLC4A2)* (g.114437192_114439942del) (Meyers et al., 2010).

The reason for DD is the single nucleotide polymorphism (SNP) g.34618072T>C in the second gene containing NHL repeats (*NHLRC2*) (OMIA 002103-9913) (Denholm et al., 2014).

The reason for M1 is an 11 bp deletion (c.821-831delTGAACACTCCA) in the third exon of myostatin gene (*MSTN*) (Aiello et al., 2018). The animals carrying in their genotypes the mutant allele had double muscling due to the muscular fiber hyperplasia and have a meat with increased tenderness. However, they also have a high weight at birth leading to dystocia (difficult calving), which destroys the reproductive functions, and so this mutation has been regarded as a genetic defect (Druet et al., 2014). In addition to Aberdeen Angus this polymorphism has also been found in some other cattle breeds particular in Belgian Blue cattle (Mota et al., 2017).

In the Hereford cattle breed the genetic defects of maple syrup urine disease (MSUD) (Dennis and Healy, 1999) and hypotrichosis (HY) (Markey et al., 2010) have been registered. The cause of MSUD is the SNP in the branched-chain keto acid dehydrogenase E1, alpha polypeptide gene (*BCKDHA*) located on chromosome 18 in the c.148C>T position. The cause of HY is the deletion of 8 bp (c.334delTGTGCCCA) in the keratin 71 (*KRT71*) gene.

The gene mutations associated with agriculturally useful traits that can beneficially affect meat productivity are also of undoubted value. One of them, the *F94L* polymorphism of the myostatin gene (*MSTN*), is the SNP in 2:g.6213980A>C position. Supposedly the generation due to the mutation of the A allele is linked to increased protein and decreased intramuscular and external fat content, which make the *F94L* polymorphism the analogue of the *nt821del MSTN* mutation caused by M1; however, in contrast to the former, the *F94L* polymorphism does not influence birth weight, and due to the absence of the dystocia risk, this polymorphism could be used for the purpose of obtaining leaner meat (Sellick et al., 2007).

The aim of our study was the analysis of the Aberdeen Angus, Hereford and Belgian Blue cattle breeds regarding the presence in the animals' genotypes of the mutant alleles associated with the appearance of the AM, OS, DD, M1, HY and MSUD genetic defects and an analysis of the frequency of the desirable A allele of the *F94L* *MSTN* polymorphism.

## 2 Material and methods

The study was conducted in 2018–2020 at the laboratory of molecular selection basis at the L.K. Ernst Federal Science Center for Animal Husbandry. The material was animals of the Aberdeen Angus (n=4473), Hereford (n=525) and Belgian Blue breeds (n=111) from farms located in the central and northwestern districts of the Russian Federation. The numbers and gender and age groups of the animals are presented in Table 1. All sampling procedures were carried out in accordance with the Federal law of 24 April 1995, N 52-FZ “On Fauna”, Article 44 “Use of the animal world for scientific, cultural, educational, recreational and aesthetic purposes”.

**Table 1:** Description of the research material.

Breed	Population${}^{*}$	Group	N
Aberdeen Angus	1a	Bulls	224
	1b	Bulls	282
	2a	Bulls	59
	2b	Bulls	91
	3	Bulls	338
	4	Bulls	3399
	5	Bulls	67
	6	Cows	20
Hereford	1a	Bulls	69
	1b	Bulls	86
	2	Bulls	190
	3	Bulls	20
	4	Cows	160
Belgian Blue	1	Cows	92

${}^{*}$ a – 2017 and b – 2019 years of birth.

From the biomaterial (skin or blood) of the animals, the DNA samples were obtained by extraction by means of the “DNA-Extran-1” and “DNA-Extran-2” kits (Syntol Company^®^ , Russia) in accordance with the manufacturer's recommendations.

The DNA samples from the animals of the Aberdeen Angus cattle breed were genotyped for the genetic defects of AM, OS and M1 by allele-specific polymerase chain reaction method (AS-PCR) and DD by PCR with the subsequent restriction fragment length polymorphism analysis (RFLP) using the restriction endonuclease *Bst MWI* (the sequence of recognition GCNNNNN↑NNGC). Genotyping was carried out by the test systems previously developed in L.K. Ernst Federal Science Center for Animal Husbandry (Konovalova et al., 2019a, 2020).

The DNA samples from the animals of the Hereford cattle breed were genotyped for the genetic defects of MSUD by a PCR-RFLP test system previously developed by Dennis et al. (1999).

Testing for HY was conducted by an AS-PCR test system developed in the laboratory of the selection molecular basis of the L.K. Ernst Federal Centre of Animal Husbandry.

A section of the animals of the Aberdeen Angus breed (n=57) and a population of the Belgian Blue breed (n=90) were studied for a mutation of the myostatin *F94L* gene by means of created in our lab PCR-RFLP test system (*TaqI* restriction endonuclease with the sequence of recognition T/CGA).

The PCR analysis was carried out on the Biometra thermocycler (Analytikjena, USA) under the following conditions: initial denaturation – 95 ${}^{\circ }$C for 3 min; 35 cycles of denaturation – 94 ${}^{\circ }$C for 45 s; annealing depending on the mutation – 55–66 ${}^{\circ }$C (Table 2) for 30 s; elongation – 72 ${}^{\circ }$C for 40 s; and final elongation – 72 ${}^{\circ }$C for 4 min. For the PCR-amplification we have used the oligonucleotide primers synthesized by EuroGene Company (Russia). The sequences presented in Table 2. The PCR mixes volumes of 10–15 µL containing 2 mM dinucleotide triphosphates mix 1 (10× PCR buffer) on 1.5–5.0 mM of each primer, 1U of *Taq*-polymerase and 1 µL PCR template.

**Table 2:** The sequences of the oligonucleotide primers for the DNA analysis of AM, OS, DD, M1, MSUD, HY and F94L mutations.

The mutation	Primer name	5${}^{\prime }$–3${}^{\prime }$ sequence	Annealing temperature, ${}^{\circ }$C
AM	AMV1	CGAAAGCCTTCTTTCCACTG	66
	AMV2	TTCTGCAGGCAAGAACACTG	
	AMV3	GAATGCCACTTCCTCCTCTG	
OS	OSF	AGCCCCTACAGTCACAGTCA	60
	OSFn	AGCAGCAGAGATCAGCTTGG	
	OSFm	CCGACCCCCTCACATTCAAA	
M1	MSTN821F	TGAGGTAGGAGAGTGTTTTGGG	60
	MSTN821Rn	CCTCTGGGGTTTGCTTGGT	
	MSTN821m	ACAGCATCGAGATTCTGTCACA	
DD	DD1	AGAGGCATGATGAAGGCGAG	61
	DD3	CCAAGGGGAACTAATGGGCT	
HY	HY_F	CGGAAGTCGGAGCCTTTACA	65
	HY_Rn	ACGCACTTTCTGGATCTCGG	
	HY_Rm	CCAGGTCAGTTGGGCACAT	
F94L	F94LF	TGAGAACAGCGAGCAGAAGG	55
	F94LR	ACTCCGTGGGCATGGTAATG	

The detection of the amplification product was conducted by electrophoresis in 3 % agarose gel under 110 V for 30 min.

After the genotyping of each genotyped population the frequency of the heterozygous genotypes (carriers) in percent was calculated by Eq. (1):

1 $$\mathrm{genotype}\;\mathrm{frequency}=\frac{N\;\text{animal carriers}}{N\;\text{population}}\times 100.$$

where N is the number of animals. For *F94L MSTN* the allele C frequency was also counted by Eq. (2):

2 $$C\;\mathrm{allele}\;\mathrm{frequency}=\frac{\text{CC}\;\mathrm{frequency}+\text{CA}\;\mathrm{frequency}}{100}.$$

The A allele frequency was counted by Eq. (3):

3 Aallelefrequency=1-Callelefrequency.

Due to the heterogeneity of the populations in size, we also calculated such an indicator as the average square deviation of the general population (Glantz 1998) using Eq. (4):

4 $$\sigma =\sqrt{\frac{\Sigma_{i=1}^{N}(x_{i}-\mu {)}^{2}}{N}},$$

where μ is the average of the general population and N is the general population size.

## 3 Results

### 3.1 DNA testing

**Figure 1:**
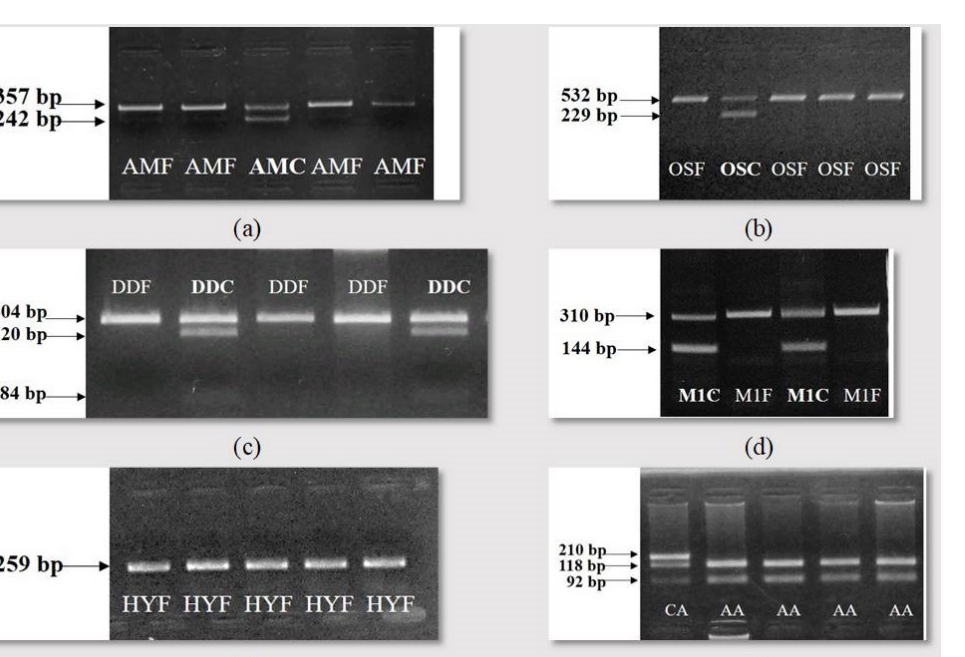
Gel electrophoresis of the amplification products: **(a)** arthrogryposis multiplex (AMF – arthrogryposis-free animal; AMC – arthrogryposis carrier); **(b)** osteopetrosis (OSF – osteopetrosis-free animal, OSC – osteopetrosis carrier); **(c)** developmental duplication (DDF – developmental duplication-free animal; DDC – developmental duplication carrier); **(d)** double muscling (M1F – double-muscling-free animal; M1C – double muscling carrier); **(e)** hypotrichosis (HYF – hypotrichosis-free animal); **(f)** genotyping for *F94L* MSTN polymorphism – presented genotypes of *CA* and *AA*.

The working of the developed test systems for the revealing of the animal carriers of the genetic defects of AM, OS, DD, M1 and HY and allele variants of the *F94L MSTN* polymorphism in the populations of Aberdeen Angus, Hereford and Belgian Blue cattle is presented in Fig. 1. The tests created allow identifying wild-type alleles and mutant ones.

### 3.2 The genetic defects in Aberdeen Angus cattle

The screening of the Russian population of Aberdeen Angus cattle has demonstrated the presence of animals carrying in their genotypes the mutant alleles associated with the appearance of the AM, DD, OS and M1 genetic defects (Table 3). AMC (arthrogryposis carrier) animals were found in three of five investigated populations with frequencies of 1.06 %–4.89 %. The average frequency was 1.84 %. OSC (osteopetrosis carrier) animals were detected in two of three investigated populations with relatively low frequencies (1.01 % and 0.67 %). The average frequency of OSC animals was 0.71 %. The average frequency of animal carriers of the M1 genetic defect was the same as OS and consisted of 0.73 %. In only one of four populations (2.1 %) were animals M1C. The highest frequency of carriers was for the DD genetic defect. DDC (developmental duplication carrier) animals were detected in five at the six investigated populations, at 1.78 %–10.4 % depending on the population. The average figure for DDC animals was 8.87 %.

**Table 3:** The frequencies of animal carriers of the genetic defects of AM, DD, OS and M1 in the Russian Aberdeen Angus populations.

Genetic defect	Population	n	Carriers %	Average frequency, %
Arthrogryposis multiplex (AM)	1a	152	0.00	1.92 ± 0.09
	1b	282	1.06	
	2a	59	0.00	
	2b	91	2.19	
	3	327	4.89	
	4	1236	1.70	
	5	36	0.00	
Developmental duplication (DD)	1a	118	0.00	9.00 ± 0.20
	1b	224	8.33	
	2a	46	0.00	
	2b	75	2.67	
	3	338	1.78	
	4	3399	10.3	
	5	67	10.4	
Osteopetrosis (OS)	1a	118	0.00	0.53 ± 0.03
	1b	190	0.00	
	2a	37	0.00	
	2b	84	0.00	
	3	296	1.01	
	4	743	0.67	
	5	37	0.00	
Double muscling (M1)	1a	32	0.00	0.19 ± 0.09
	1b	280	0.00	
	2a	40	0.00	
	2b	73	0.00	
	3	22	0.00	
	4	46	2.10	
	5	37	0.00	

### 3.3 The genetic defects in Hereford cattle

The genotyping of the Russian population of Hereford cattle did not find the animal carriers of the HY and MSUD genetic defects.

### 3.4 The genotyping of the Belgian Blue cattle population for the M1 genetic defect

**Figure 2:**
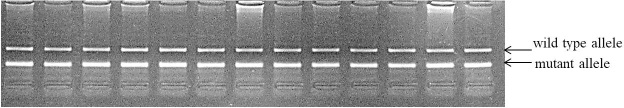
The fragment of the genotyping of the Belgian Blue population for the M1 genetic defect.

Because the *nt821del11 MSTN* polymorphism was also registered in the Belgian Blue cattle breed, we also genotyped the population of this breed for this mutation. As a result, we found that the absolute majority of the investigated animals showed the pattern in Fig. 2 on the electrophoresis and were carriers of the M1 genetic defect, having in their genotypes the wild-type allele (310 bp) and the mutant one (144 bp). This fact can be explained by the fixation of the mutant allele due to the long-term selection for increased muscle mass in the past (Mota et al., 2017).

### 3.5 F94L polymorphism genotyping

The study of the *F94L* polymorphism showed high frequencies in the two populations (3 and 6) of Aberdeen Angus cattle of the desirable A allele: 0.93 and 0.90, respectively. This fact is probably one of the explanations of the easy calving observed in the animals of this breed (Dyuldina, 2016). The average A allele frequency in the investigated Aberdeen Angus population was 0.63, which was 0.32 (32 %) higher than in the Belgian Blue one (Table 4). But the Belgian Blue population was more polymorphic compared to the Aberdeen Angus one because there were animals with three possible genotypes for the *F94L MSTN* polymorphism: *CC* – 20.6 %; *CA* – 48.9 %; *AA* – 30.5 %.

**Table 4:** The results of the genotyping of Aberdeen Angus and Belgian Blue cattle for the *F94L* *MSTN* polymorphism.

Breed	Population	n	Genotype			Allele	
			frequency, %			frequency	
			*CC*	*CA*	*AA*	C	A
Aberdeen Angus	1b	18	94.4	5.6	0.0	0.94	0.06
	3	29	0.0	6.9	93.1	0.07	0.93
	6	20	0.0	10.0	90.0	0.10	0.90
Belgian Blue	1	92	20.6	48.9	30.5	0.69	0.31

## 4 Discussion

### 4.1 The development of the test systems for the genetic defects diagnostics in Russia

It is well known that the best method for the exact identification of the mutant alleles linked with genetic disorders such as productivity traits is DNA analysis. This provides the possibility of getting data about gene mutations independently of the gender, age and physiological status of the animal. Previously, DNA tests were developed which were based on the PCR strategy and directed at revealing mutant alleles associated with genetic defects in cattle genotypes in the Aberdeen Angus and Hereford cattle breeds (Beever et al., 2014; Dennis et al., 1999; Markey et al., 2010; Mayers et al., 2010). Until 2017, in Russia, the problem of meat cattle genetic disorders had not been study in detail and the tools for revealing the latent carriers of some genetic defects were absent. Only in 2019–2020, based on the data from Online Mendelian Inheritance in Animals (OMIA) and the National Center Biotechnology Information, was the test system for diagnostics of the AM, OS, DD and M1 genetic defects characteristic of the Aberdeen Angus breed and the MSUD and HY characteristic of the Hereford breed using AS-PCR and PCR-RFLP methods created (Konovalova et al., 2019a, 2020). These achievements are very important for the domestic meat breeding industry because they provide the possibility of testing meat cattle and their breeding material on Russian territory.

The modern development of molecular biology gives a good possibility of speedy gene diagnostics by using the method of real-time PCR. Indeed, this strategy is more appropriate for high-throughput genotyping compared to the end-point PCR diagnostics. However, in our opinion, it is not entirely reasonable to refuse to use the traditional PCR methods, since any molecular genetic laboratory should have various diagnostic tools at its disposal. The development of test systems based on AS-PCR and PCR-RFLP methods, the main advantage of which is less dependence on the computer software, is the initial stage of work on the problem, and further we do not exclude the modernization of these methods by the most modern molecular genetic techniques.

### 4.2 The genetic load evaluation of the Russian cattle populations of the Aberdeen Angus, Hereford and Belgian Blue breeds

The screening of the investigated Aberdeen Angus cattle for the genetic defects of AM, OS, DD and M1 mainly revealed separate animal carriers of the genetic defects in some populations. Some populations were free of the mutations causing AM, OS, DD and M1. Relating to the AM, OS and M1 genetic defects, the frequencies of animal carriers did not exceed 5 %. The greatest concern regards the genetic defect of developmental duplication: in populations 4 and 5, the frequency of the occurrence of animals carrying the defect was 10.3 % and 10.4 % (Table 3).

The absence in the studied Hereford populations of animal carriers of the genetic defects of HY and MSUD was a pleasant surprise for us and is a very favorable sign.

It is notable that all animals of the investigated Belgian Blue cattle were carriers of the mutant allele associated with the M1 genetic defect. The mutant allele frequency was 0.5. This fact can be in part explained by the history of breeding Belgian Blue cattle. At the beginning of the twentieth century, the breed was widespread in southern Belgium and used to be a dual-purpose breed called the “Mid and Upper Belgium Breed”. However, from the 1950s to the 1980s, beef selection by breeders led to an unintentional fixation of the mutant allele associated, as was clarified in 1997 (McPherron et al., 1997), with mh (muscular hypertrophy) (Druet et al., 2014; Mota et al., 2017). On the one hand, the mh allele is associated with higher meat yield and more tender meat but on the other hand with an enlarged birth weight of calves and, linked to this, calving difficulty, which requires a Cesarean section. This makes this trait a genetic defect that limits the breeding of Belgian Blue cattle (Mota et al., 2017).

### 4.3 The justification of the need for control over the genetic defects in meat cattle breeding

Despite the fact of the absence of individual carriers of genetic defects in the some investigated populations, we are sure that control of the cattle genetic defects is necessary independently of the breed due to the existence of the risk of gene mutation spread. Previously, we have already observed on the farm the absence of animal carriers of the AM and DD genetic defects in 2015 and the presence of these defects in 2019 (2.73 % AMC and the same frequency for DDC) (Konovalova et al., 2019b).

It should be noted that state regulation of the problem by the foundation of breed associations is important. Along with the availability of data on animal productivity, the most solid associations have data on animals that carry inherited abnormalities obtained through DNA analysis. The rules for the use of such animals and their breeding material are also regulated. These measures prevent the spread of genetic defects and clinical cases of the inherited diseases.

### 4.4 The *F94L* MSTN genotyping displayed the existence of meat productivity potential in the Russian populations of Aberdeen Angus and Belgian Blue cattle

The identification of genes controlling production traits in farm animal species will lead to cost-effective genetic testing of entire populations for the enhanced selection of individuals, thus providing increased product quality and production efficiency.

Sellick et al. (2007) stated that “A previously reported transversion in *MSTN* (AF320998.1, g.433C>A), resulting in the amino acid substitution of phenylalanine by leucine at position 94 of the protein sequence (F94L), was the only polymorphism consistently related to increased muscling. Overall the size of the g.433C>A additive effect on carcass traits was moderately large, with the g.433A allele found to be associated with a 5.5 % increase in silverside percentage and eye muscle area (EMA) and a 2.3 % increase in total meat percentage relative to the g.433C allele. The phenotypic effects of the g.433A allele were partially recessive”. Probably the *MSTN* genotype can produce an intermediate, non-double-muscling phenotype, which should be of significant value for beef cattle producers (Sellick et al., 2007).

The result of the genotyping of the Aberdeen Angus and Belgian Blue populations in our study has shown the presence of the desirable A allele *F94L* *MSTN* polymorphism in the both breeds. The frequency of the A allele was higher in the two Aberdeen Angus populations (0.90 and 0.93) compared with the Belgian Blue one (0.31). However, despite the presence of the some mutant alleles associated with genetic defects, the *F94L* genotyping showed the existence of genetic potential for the productivity traits in the cattle of the Aberdeen Angus and Belgian Blue breeds.

## 5 Conclusions

The obtained DNA analysis data have demonstrated the high genetic potential of the Aberdeen Angus and Belgian Blue cattle due to enough high frequencies of desirable variants of the *F94L* polymorphism. The use of this mutation as a genetic marker would be able to provide the possibility of obtaining leaner meat and avoiding the problems with animal health due to the absence of the influence on calf weight at birth and its association with dystocia.

Taking into account the presence in the populations of Aberdeen Angus cattle of animal carriers of the AM, OS, DD and M1 genetic defects, the constant control for congenital anomalies in Aberdeen Angus herds by means of DNA genotyping is necessary.

The results showed the absence in the Hereford populations of individuals carrying the HY and MSUD genetic defects; this is a very good sign, making it possible to use this potential breed without strict limitations.

The results of this research allow us to conclude that the breeding of Aberdeen Angus, Hereford and Belgian Blue cattle with proper management has good potential for improving meat cattle industry profitability.

## Data Availability

The original data of the paper are available upon request from the corresponding author.

## References

[bib1.bib1] Aiello D, Patel K, Lasagna E (2018). The myostatin gene: an overview of mechanisms of action and its relevance to livestock animals. Anim Genet.

[bib1.bib2] Beever JE, Marron BM, Parnell PF, Teseling CF, Steffen DJ, Denholm LJ (2014). Developmental Duplications (DD): 1. Elucidation of the underlying molecular genetic basis of polymelia phenotypes in Angus cattle.

[bib1.bib3] Denholm LJ, Martin LE, Teseling CF, Parnell PF, Beever JE (2014). Developmental Duplications (DD): 2. Mutation of the NHLRC2 gene causes neural tube defects in Angus cattle with multiple congenital malformation phenotypes that include axial and limb duplications, heteropagus conjoined twins, midbrain and forebrain malformations including pseudoholoprosencephalon, craniofacial dysmorphogenesis, micropthalmia, diprosopus, embryogenic teratomas, dermoid cysts, myolipomas, split cord malformation and cranial and spinal dysraphism.

[bib1.bib4] Dennis JA, Healy PJ (1999). Definition of the mutation responsible for maple syrup urine disease in poll shorthorns and genotyping poll shorthorns and poll Herefords for maple syrup urine disease alleles. Res Vet Sci.

[bib1.bib5] Druet T, Ahariz N, Cambisano N, Tamma N, Michaux C, Coppieters W, Charlier C, Georges M (2014). Selection in action: dissecting the molecular underpinnings of the increasing muscle mass of Belgian blue cattle. BMC Genom.

[bib1.bib6] Dusaeva EM, Kuvanov ZN (2013). Statistical study of the world beef market. News of the Orenburg state agrarian University.

[bib1.bib7] Dyuldina AV (2016). Meat productivity of steers Aberdeen Angus breed of different origin. Dairy and beef cattle farming.

[bib1.bib8] Gholap PN, Kale DS, Sirothia AR (2014). Genetic Diseases in Cattle: A Review. Res J Anim Vet Fish Sci.

[bib1.bib9] Glantz SA (1998). Primer of biostatistics.

[bib1.bib10] Konovalova EN, Kostyunina OV, Zinovieva NA (2019). Diagnostic technique for polymorphism of genes AGRN, ISG15 and HES4 causing lethal genetic defect of multiple arthrogryposis of cattle meat breeds, RU Patent 2703396C2.

[bib1.bib11] Konovalova EN, Kostiunina OV, Romanenkova OS (2019). Aberdeen Angus cattle breed in Russia: prevention of the genetic defects and evaluation of the risk of their spread by transferring from parents to offspring. proceedings of IOP Conf Ser: Earth Environ Sci Krasnoyarsk.

[bib1.bib12] Konovalova EN, Kostyunina OV, Zinovieva NA (2020). Method of diagnosing polymorphism of NHLRC2 gene causing genetic defect of duplication of aberdeen-angus breed cattle development, RU Patent 2715330C2.

[bib1.bib13] Markey AD, Taylor JF, Schnabel RD, McKay SD, McClure MC, Beever JE (2010). A deletion mutation in KRT71 is associated with congenital hypotrichosis in Hereford cattle. proceedings of the Plant & Animal Genomes XVIII Conference.

[bib1.bib14] McPherron AC, Lawler AM, Lee S-J (1997). Regulation of skeletal muscle mass in mice by a new TGF-beta superfamily member. Nature.

[bib1.bib15] Meyers SN, McDaneld TG, Swist SL, Marron BM, Steffen DJ, O'Toole D, O'Connell JR, Beever JE, Sonstegard TS, Smith TP (2010). A deletion mutation in bovine SLC4A2 is associated with osteopetrosis in Red Angus cattle. BMC Genom.

[bib1.bib16] Mota RR, Mayeres P, Bastin C, Glorieux G, Bertozzi C, Vanderick S, Hammami H, Colinet FG, Gengler N (2017). Genetic evaluation for birth and conformation traits in dual-purpose Belgian Blue cattle using a mixed inheritance model. J Anim Sci.

[bib1.bib17] Ragimov GI, Zhuchaev KV, Kochneva ML, Gart VV, Inerbaev BO, Goncharenko GM, Deeva VS (2019). Hereford and Simmental Cattle Breeds in Siberia: Implementation of the Adaptive and Productive Potential in the Cold Climate. IJRTE.

[bib1.bib18] Sellick GS, Pitchford WS, Morris CA, Cullen NG, Crawford AM, Raadsma HW, Bottema CD (2007). Effect of myostatin F94L on carcass yield in cattle. Anim Genet.

[bib1.bib19] Urioste JI, Misztal I, Bertrand JK (2007). Fertility traits in spring-calving Aberdeen Angus cattle. 1. Model development and genetic parameters. J Anim Sci.

[bib1.bib20] Windsor P, Kessell A, Finnie J (2011). Neurological diseases of ruminant livestock in Australia. V: congenital neurogenetic disorders of cattle. Aust Vet J.

